# Artificial intelligence model for segmentation and severity scoring of osteophytes in hand osteoarthritis on ultrasound images

**DOI:** 10.3389/fmed.2024.1297088

**Published:** 2024-03-04

**Authors:** Benjamin Schultz Overgaard, Anders Bossel Holst Christensen, Lene Terslev, Thiusius Rajeeth Savarimuthu, Søren Andreas Just

**Affiliations:** ^1^Section of Rheumatology, Department of Medicine, Svendborg Hospital – Odense University Hospital, Svendborg, Denmark; ^2^ROPCA ApS, Odense, Denmark; ^3^Center for Rheumatology and Spine Disease, Rigshospitalet, Glostrup, Denmark; ^4^Mærsk Mc-Kinney Møller Institute, University of Southern Denmark, Odense, Denmark

**Keywords:** osteoarthritis, artificial intelligence, robotics, automated ultrasound scanning, neural networks, hand osteoarthritis, osteophyte

## Abstract

**Objective:**

To develop an artificial intelligence (AI) model able to perform both segmentation of hand joint ultrasound images for osteophytes, bone, and synovium and perform osteophyte severity scoring following the EULAR-OMERACT grading system (EOGS) for hand osteoarthritis (OA).

**Methods:**

One hundred sixty patients with pain or reduced function of the hands were included. Ultrasound images of the metacarpophalangeal (MCP), proximal interphalangeal (PIP), distal interphalangeal (DIP), and first carpometacarpal (CMC1) joints were then manually segmented for bone, synovium and osteophytes and scored from 0 to 3 according to the EOGS for OA. Data was divided into a training, validation, and test set. The AI model was trained on the training data to perform bone, synovium, and osteophyte identification on the images. Based on the manually performed image segmentation, an AI was trained to classify the severity of osteophytes according to EOGS from 0 to 3. Percent Exact Agreement (PEA) and Percent Close Agreement (PCA) were assessed on individual joints and overall. PCA allows a difference of one EOGS grade between doctor assessment and AI.

**Results:**

A total of 4615 ultrasound images were used for AI development and testing. The developed AI model scored on the test set for the MCP joints a PEA of 76% and PCA of 97%; for PIP, a PEA of 70% and PCA of 97%; for DIP, a PEA of 59% and PCA of 94%, and CMC a PEA of 50% and PCA of 82%. Combining all joints, we found a PEA between AI and doctor assessments of 68% and a PCA of 95%.

**Conclusion:**

The developed AI model can perform joint ultrasound image segmentation and severity scoring of osteophytes, according to the EOGS. As proof of concept, this first version of the AI model is successful, as the agreement performance is slightly higher than previously found agreements between experts when assessing osteophytes on hand OA ultrasound images. The segmentation of the image makes the AI explainable to the doctor, who can immediately see why the AI applies a given score. Future validation in hand OA cohorts is necessary though.

## 1 Introduction

Hand osteoarthritis (OA) is a common condition with a lifetime risk of symptomatic hand OA of 40% (1). Symptoms of hand OA are pain, stiffness and loss of normal joint function and are associated with a decrease in quality of life (2). Hand OA further leads to impairment in work participation, which results in substantial societal costs of lost productivity (3). Hand OA is a heterogeneous disease, with ultrasound findings as osteophytes, joint effusion, synovial hypertrophy, inflammation, and joint space narrowing (4).

Greyscale ultrasound of finger joints has been proven to be a reliable and sensitive method for the detection of osteophytes in patients with hand OA (5).

A semiquantitative grading system from 0 to 3 has been developed and validated to describe the severity of osteophytes in hand OA (6–8). The EULAR-OMERACT grading system (EOGS) for osteophytes creates a potential for precise osteophyte detection and monitoring using ultrasound (8). However, a thorough ultrasound examination, image analysis and scoring require an experienced professional and is time-consuming.

A new automated system has been developed to perform a quality ultrasound examination of the hands without needing a trained professional (9). The ARTHUR system can detect inflammatory arthritis in finger joints and wrist and score severity through AI (9–11). However, it cannot currently detect and grade osteophytes in hand OA. An automated method of detecting and grading hand OA could benefit clinical practice and future trials.

Artificial intelligence (AI) has been widely recognized as a technology that will affect many industries, including the health sector. Rheumatology and ophthalmology are just two areas of the health sector which will be affected by the technology (12, 13). With the help of clinical experts for the generation and annotation of high-quality data and by translating their clinical knowledge into AI systems, it is possible to develop automated diagnosis and decision support systems.

AI development for interpreting ultrasound images for the different hallmarks of hand OA is progressing. In joint space narrowing, AI models measuring metacarpophalangeal (MCP) cartilage thickness, have been presented (14, 15). Within inflammation assessment of hand joints, the models in the literature are primarily developed using RA patients. They show that developing AI for detecting and grading arthritis on ultrasound images is possible (10, 16). Within the field of AI models for osteophyte assessment, we did not find any previous published work. This study therefore aimed to develop, as a proof of concept, an AI model capable of grading osteophytes according to the OA EOGS, with a performance comparable to grading between human experts.

## 2 Materials and m**ethods**

### 2.1 Study design

One hundred sixty patients from the Section of Rheumatology at Svendborg Hospital, Odense University Hospital, with hand pain or reduced hand function were included. Patients were asked to participate during planned outpatient clinic visits from January to April 2023. Patients are therefore a mix of patients coming to monitoring of existing inflammatory disease, and new patients referred due to a suspicion of inflammatory disease. Patients with severe joint deformations were excluded. The protocol was evaluated by both the local ethics committee (S-20222000–136, 25. Nov. 2022) and the National Research Ethics Medical Committee (KBJ correspondence, 10. Nov. 2022) for acceptance and reporting obligations, and both determined that the study did not meet the criteria to need their approval. The protocol was registered as a quality project by Odense University Hospital (OUH) (22/60212, 20. Dec. 2022). All patients signed informed consent for participation.

### 2.2 Image protocol and analysis

An ultrasound scan of both hands was performed with a General Electric (GE, Chicago, Illinois, USA) Logiq E10 with a GE ML 6–15 probe. Greyscale pictures were obtained of the metacarpophalangeal (MCP), proximal interphalangeal (PIP), distal interphalangeal (DIP) and first carpometacarpal (CMC) joints in the longitudinal plane from the dorsal side with the joint centered. For each patient, 30 ultrasound pictures (10 MCP, 10 PIP, 8 DIP and 2 CMC) were manually segmented into bone, synovium and osteophytes using the open source software CVAT (17). All images and segmentations were then assessed for quality by a rheumatologist, and the pictures were subsequently scored for osteophyte severity from 0 to 3 according to the EOGS (8). The rheumatologist assessing for quality has over 10 years’ experience in musculoskeletal ultrasound, has published in the field and is a frequent teacher and organizer of musculoskeletal ultrasound courses.

The total number of images obtained for AI development is shown in Table 1.

**TABLE 1:** Data generated for the AI development.

Joint	MCP	PIP/IP	DIP	CMC	Total
Images	1599	1598	1218	320	4734
Removed due to missing ground truth (%)	24 (1,5)	22 (1,4)	21 (1,7)	11 (3,4)	78 (1,6)
Removed due to quality* (%)	15 (0,9)	17 (1,1)	3 (0,2)	7 (2,2)	42 (0,9)
Total images used for AI development (%)	1560 (97,6)	1559 (97,6)	1194 (98,0)	302 (96,4)	4615 (97,5)

*Excluded by a clinical expert in rheumatology and ultrasound.

### 2.3 Data preparation

Before training the AI model, the data was divided into three datasets: training, validation, and testing. The training set contained 80% of the data (3,693 images). The validation- and test set contained 10% of the total data, respectively (461 images). Each image was randomly sampled into one of the three datasets. After the datasets had been generated, it was verified that the distribution of joints was similar in the three datasets.

The training set was used for training the AI algorithms. The images, annotations and ground truth grading in this dataset directly influenced the updating of the model weights. The validation set was used to validate the model’s performance on separate data during training, but the validation set was not used to train the algorithm directly. During the development of the AI algorithm, configurations were made to optimize the performance of the validation set. The test set was only used for performance evaluation after all configuration settings had been made.

Before training, the data was normalized such that all image pixels were in the range [−1: +1] as is normal procedure for data for training AI, and data augmentation was applied to artificially enhance the data in the dataset by applying realistic manipulations on the pixels of the images. For ultrasound images of the finger joints, this includes realistic pixel manipulations such as magnification, rotation and variations of brightness in the images.

### 2.4 Development of AI for segmentation

A convolutional neural network architecture called U-Net++ (18) was trained to identify and mark, also called segment, bones, synovia and osteophytes on b-mode ultrasound images of the finger joints. The U-Net++ is a more robust architecture than the widely known U-Net architecture and is designed specifically for medical image segmentation. Compared to U-Net, U-Net++ adds connections from the encoder to the decoder in the network for more precise segmentation results. The model has a total of 36,157,321 trainable weights.

### 2.5 Statistical analyses

With the expert sonographer score as the gold standard, the percentage of exact agreement (PEA) and percentage of close agreement (PCA) were calculated for OA scoring. The PEA was calculated for all grades (0–3). The PCA was defined as the percentage of the patients where the scores differed by no more than 1. In addition, the sensitivity and specificity of the AI model were calculated with dichotomized EOGS scores considering grade 0 absence and grades 1–3 as presence of osteophytes.

## 3 Results

Baseline patient characteristics are presented in Table 2. The performance of the developed AI model, on 0–3 osteophyte scoring according to the EOGS, divided into joints, is presented in Table 3. In the same table, results of AI assessment of the validation and test set are presented. A complete presentation of these results for the test set, including confusion matrixes, is presented in Supplementary Table 1.

**TABLE 2:** Patient characteristics.

Patient characteristics (*n* = 160)	
Age (years, mean ± SD)	61,6 ± 14,6
Female (n,%)	110 (69%)
**Rheumatological diagnosis**	
Rheumatoid arthritis (n,%)	91 (57%)
Psoriatic arthritis (n,%)	18 (11%)
Unspecified arthritis (n,%)	5 (3,1%)
Axial spondylarthritis (n,%)	5 (3,1%)
Polymyalgia rheumatica (n,%)	4 (2,5%)
Other or no rheumatic diagnosis (n,%)	37 (23%)

**TABLE 3:** Precision of AI on 0–3 OA scoring using the rheumatologist score as gold standard.

Dataset	Joints	PEA (%)	PCA (%)	Sensitivity (%)	Specificity (%)
Validation	All joints	75.05	95.01	52.90	89.12
	MCP	83.44	100.00	63.33	91.74
	PIP	73.73	91.82	48.58	87.90
	DIP	72.73	95.04	38.89	92.94
	CMC	61.54	89.74	70.00	78.95
Test	All joints	68.11	95.23	50.42	81.87
	MCP	75.76	96.97	48.65	85.94
	PIP	70.27	96.62	46.67	83.90
	DIP	59.17	94.17	59.09	77.63
	CMC	50.00	82.14	25.00	60.00

PEA, Percent Exact Agreement; PCA, Percent Close Agreement; MCP, Metacarpophalangeal; PIP, Proximal Interphalangeal; DIP, Distal Interphalangeal; CMC, first carpometacarpal joint.

**FIGURE 1:**
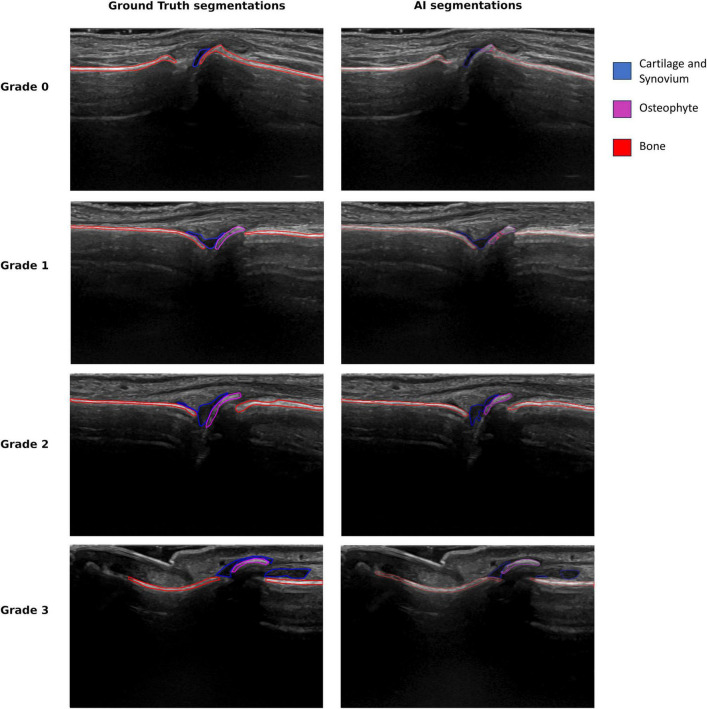
Ground truth and AI segmentation on an example image from each EOGS score.

Examples of the segmentation capabilities of the developed AI model are presented in Figure 1. The AI marks bone as red, synovium, including cartilage as blue and osteophytes as pink.

## 4 Discussion

This is the first time an AI model has been developed for segmentation and semiquantitative scoring of osteophytes on ultrasound images following the EOGS.

We demonstrate that the PEA between AI and experts was slightly higher than between experts in previous studies (6, 7). Here, PEA for EOGS osteophyte 0–3 scoring was 54.2% and 61%, respectively, while PEA in this study was 75.1% in the validation set and 68.1% in the test set. This suggests that the developed AI model is a success as a proof of concept, showing that AI can potentially be a viable method for osteophyte assessment on ultrasound images.

Segmentation of the image, as seen in Figure 1, is essential, as it explains to the healthcare professional how the AI model has interpreted the ultrasound image and reached its conclusion of the given OA grade in the joint. It does this by marking on the image the location and size of the bone part it regards as an osteophyte. This contrasts with earlier AI models in other diseases, which could be described as “black box” methods, e.g., only giving a score. Explainable AI is essential for developing systems that medical professionals trust. Further, it is also a vital part of the process of CE marking medical AI imaging systems by the European Union’s medical device regulations (EU MDR).

The AI is developed to grade osteophytes according to the EOGS. The EULAR OMERACT grading system provides a standardized framework for assessing osteophytes, enabling consistent and reproducible measurements across clinical settings. The use of this system ensures that our AI algorithm’s performance can be directly compared to previous findings and that it is an internationally accepted standard.

One of the primary limitations of this study is that the majority of patients included in this study have inflammatory arthropathies, especially RA (see Table 1). Patients with these diseases can also have hand OA, and joint osteophytes, as can be seen in this study. Going forward to further develop and validate the algorithm a cohort of only hand OA patient will be assessed. Another limitation is the use of one expert to define ground truth. Future development of the model will include more images scored by different experts. AI for ultrasound analysis does not replace the need for clinical evaluation but has several strengths when applied. In addition, the model can be further developed and trained with more images, which is currently ongoing.

Another aspect, outside the scope of this study, for future developments of the automated scanning system, is to assess osteophyte severity in other probe positions than the standard position. Performing sweeps over the joint while collecting and assessing images continuously, could possibly detect joint disease outside the EOGS standard position.

The presented AI model segments cartilage as part of the synovium (marked blue on the images). The images obtained in this study were scanned according to OA osteophyte evaluation and EOGS OA scoring (7). Cartilage thickness in hand OA is recommended to be assessed with maximal flexion, e.g., the MCP joint scanned with a high-frequency hockey stick probe (7). This was not done in this study. As cartilage abnormalities are a part of the hand OA pathogenesis, this could be interesting to include in our future OA AI model development. Previous research has demonstrated the feasibility of developing AI models for measuring cartilage thickness, particularly when utilizing high-frequency probes for targeted image acquisition (14, 15).

Taking a step back looking at the situation in AI development for hand joint ultrasound assessment, models have been created targeting different aspects that can be seen in hand OA. These are cartilage thickness assessment, inflammation with arthritis assessment, and with this publication osteophyte assessment. Going forward, developing a unifying AI model combining all traits, and thereafter training and validating this on hand OA patients would be a marked improvement. This could open up for a much more detailed understanding of the very heterogenous disease hand OA, how these factors interact, and change over time. This unified hand OA AI model could thereby potentially also assist in the stratification of hand OA patients for clinical trials, used in monitoring during the trial, and possibly enable more targeted therapies against hand OA.

## Data availability statement

The original contributions presented in this study are included in the article/Supplementary material, further inquiries can be directed to the corresponding author.

## Ethics statement

The requirement of ethical approval was waived by the De Videnskabsetiske Komiteér for Region Syddanmark (VEK) De Videnskabsetiske Medicinske Komitéer (VMK) for the studies involving humans. The studies were conducted in accordance with the local legislation and institutional requirements. The participants provided their written informed consent to participate in this study.

## Author contributions

BO: Conceptualization, Data curation, Methodology, Project administration, Visualization, Writing−original draft, Writing−review and editing. AC: Conceptualization, Data curation, Formal Analysis, Project administration, Software, Writing−original draft, Writing−review and editing. LT: Conceptualization, Methodology, Supervision, Writing−review and editing. TS: Conceptualization, Methodology, Supervision, Validation, Writing−review and editing. SJ: Conceptualization, Formal Analysis, Funding acquisition, Investigation, Methodology, Project administration, Supervision, Writing−original draft, Writing−review and editing.

## References

[1] Qin J Barbour K Murphy L Nelson A Schwartz T Helmick C Lifetime risk of symptomatic hand osteoarthritis: The Johnston county osteoarthritis project. *Arthritis Rheumatol.* (2017) 69:1204–12. 10.1002/art.40097 28470947 PMC5449255

[2] Pathmanathan C Deveza L Robbins S Duong V Venkatesha V Hunter D Determinants of quality of life and hand function among people with hand osteoarthritis. *Int J Rheum Dis.* (2022) 25:1408–15. 36086872 10.1111/1756-185X.14435

[3] Terpstra SES van de Stadt L Boonen A Damman W Rosendaal F Kloppenburg M Hand osteoarthritis is associated with limitations in paid and unpaid work participation and related societal costs: The HOSTAS cohort. *RMD Open.* (2022) 8:e002367. 10.1136/rmdopen-2022-002367 PMC9345053 35906024

[4] Kloppenburg M Kwok WY. Hand osteoarthritis–a heterogeneous disorder. *Nat Rev Rheumatol.* (2011) 8:22–31. 22105244 10.1038/nrrheum.2011.170

[5] Mathiessen A Haugen I Slatkowsky-Christensen B Bøyesen P Kvien T Hammer H Ultrasonographic assessment of osteophytes in 127 patients with hand osteoarthritis: Exploring reliability and associations with MRI, radiographs and clinical joint findings. *Ann Rheum Dis.* (2013) 72:51–6. 10.1136/annrheumdis-2011-201195 22523427

[6] Keen HI Lavie F Wakefield R D’Agostino M Hammer H Hensor E The development of a preliminary ultrasonographic scoring system for features of hand osteoarthritis. *Ann Rheum Dis.* (2008) 67:651–5. 10.1136/ard.2007.077081 17704062

[7] Hammer HB Iagnocco A Mathiessen A Filippucci E Gandjbakhch F Kortekaas M Global ultrasound assessment of structural lesions in osteoarthritis: A reliability study by the OMERACT ultrasonography group on scoring cartilage and osteophytes in finger joints. *Ann Rheum Dis.* (2016) 75:402–7. 10.1136/annrheumdis-2014-206289 25520476

[8] Mathiessen A Hammer H Terslev L Kortekaas M D’Agostino M Haugen I Ultrasonography of inflammatory and structural lesions in hand osteoarthritis: An outcome measures in rheumatology agreement and reliability study. *Arthritis Care Res.* (2022) 74:2005–12. 10.1002/acr.24734 34137211

[9] Frederiksen BA Schousboe M Terslev L Iversen N Lindegaard H Savarimuthu T Ultrasound joint examination by an automated system versus by a rheumatologist: From a patient perspective. *Adv Rheumatol.* (2022) 62:30. 10.1186/s42358-022-00263-2 35941629

[10] Christensen ABH Just S Andersen J Savarimuthu T. Applying cascaded convolutional neural network design further enhances automatic scoring of arthritis disease activity on ultrasound images from rheumatoid arthritis patients. *Ann Rheum Dis.* (2020) 79:1189–93. 10.1136/annrheumdis-2019-216636 32503859

[11] Andersen JKH Pedersen J Laursen M Holtz K Grauslund J Savarimuthu T Neural networks for automatic scoring of arthritis disease activity on ultrasound images. *RMD Open.* (2019) 5:e000891. 10.1136/rmdopen-2018-000891 PMC6443126 30997154

[12] McMaster C Bird A Liew D Buchanan R Owen C Chapman W Artificial intelligence and deep learning for rheumatologists. *Arthritis Rheumatol.* (2022) 74:1893–905. 35857865 10.1002/art.42296 PMC10092842

[13] Gulshan V Peng L Coram M Stumpe M Wu D Narayanaswamy A Development and validation of a deep learning algorithm for detection of diabetic retinopathy in retinal fundus photographs. *JAMA.* (2016) 316:2402–10. 27898976 10.1001/jama.2016.17216

[14] Fiorentino MC Cipolletta E Filippucci E Grassi W Frontoni E Moccia S. A deep-learning framework for metacarpal-head cartilage-thickness estimation in ultrasound rheumatological images. *Comput Biol Med.* (2022) 141:105117. 10.1016/j.compbiomed.2021.105117 34968861

[15] Cipolletta E Fiorentino M Moccia S Guidotti I Grassi W Filippucci E Artificial intelligence for ultrasound informative image selection of metacarpal head cartilage. A pilot study. *Front Med.* (2021) 8:589197. 10.3389/fmed.2021.589197 33732711 PMC7956959

[16] Wu M Wu H Wu L Cui C Shi S Xu J A deep learning classification of metacarpophalangeal joints synovial proliferation in rheumatoid arthritis by ultrasound images. *J Clin Ultrasound.* (2022) 50:296–301. 10.1002/jcu.23143 35038176

[17] MIT. *Computer vision annotation Tool (CVAT).* (2023). Available online at: https://github.com/opencv/cvat (accessed September 2, 2023).

[18] Zhou Z Siddiquee M Tajbakhsh N Liang J. UNet++: A nested u-net architecture for medical image segmentation. *Deep Learn Med Image Anal Multimodal Learn Clin Decis Support (2018).* (2018) 11045:3–11. 10.1007/978-3-030-00889-5_1 32613207 PMC7329239

